# Morphogenetic fields within the human dentition: A new, clinically relevant synthesis of an old concept^☆^

**DOI:** 10.1016/j.archoralbio.2008.06.011

**Published:** 2009-12

**Authors:** Grant Townsend, Edward F. Harris, Herve Lesot, Francois Clauss, Alan Brook

**Affiliations:** a School of Dentistry, The University of Adelaide, Adelaide, South Australia 5005, Australia; b Department of Orthodontics, College of Dentistry, University of Tennessee, 875 Union Avenue, Memphis, TN 38163, USA; c INSERM U595, Faculty of Medicine, 11, rue Humann, 67085 Strasbourg, France; d Faculty of Dentistry, Louis Pasteur University, 67085 Strasbourg Cedex, France; e International Collaborating Centre in Oro-facial Genetics and Development, The University of Liverpool, Liverpool L69 3GN, UK

**Keywords:** Dental development, Clone model, Homeobox code, Patterning, Hypodontia, Supernumeraries, Twins

## Abstract

This paper reviews the concept of morphogenetic fields within the dentition that was first proposed by Butler (Butler PM. Studies of the mammalian dentition. Differentiation of the post-canine dentition. *Proc Zool Soc Lond B* 1939;**109**:1–36), then adapted for the human dentition by Dahlberg (Dahlberg AA. The changing dentition of man. *J Am Dent Assoc* 1945;**32**:676–90; Dahlberg AA. The dentition of the American Indian. In: Laughlin WS, editor. *The Physical Anthropology of the American Indian*. New York: Viking Fund Inc.; 1951. p. 138–76). The clone theory of dental development, proposed by Osborn (Osborn JW. Morphogenetic gradients: fields versus clones. In: Butler PM, Joysey KA, editors *Development, function and evolution of teeth*. London: Academic Press, 1978. p. 171–201), is then considered before these two important concepts are interpreted in the light of recent findings from molecular, cellular, genetic and theoretical and anthropological investigation. Sharpe (Sharpe PT. Homeobox genes and orofacial development. *Connect Tissue Res* 1995;**32**:17–25) put forward the concept of an odontogenic homeobox code to explain how different tooth classes are initiated in different parts of the oral cavity in response to molecular cues and the expression of specific groups of homeobox genes. Recently, Mitsiadis and Smith (Mitsiadis TA, Smith MM. How do genes make teeth to order through development? *J Exp Zool (Mol Dev Evol)* 2006; **306B**:177–82.) proposed that the field, clone and homeobox code models could all be incorporated into a single model to explain dental patterning. We agree that these three models should be viewed as complementary rather than contradictory and propose that this unifying view can be extended into the clinical setting using findings on dental patterning in individuals with missing and extra teeth. The proposals are compatible with the unifying aetiological model developed by Brook (Brook AH. A unifying aetiological explanation for anomalies of tooth number and size. *Archs Oral Biol* 1984;**29**:373–78) based on human epidemiological and clinical findings. Indeed, this new synthesis can provide a sound foundation for clinical diagnosis, counselling and management of patients with various anomalies of dental development as well as suggesting hypotheses for future studies.

## 1 Field and clone theories

The concept of a morphogenetic field emerged in the 19th century, though the molecular-genetic basis for patterned series of skeletodental elements (e.g., vertebrae, ribs, teeth) were unknown. Ordered form was a common observation in numerous aspects of biology,^8^ but the governing principles, let alone the causes, were virtually unknown.^9^

The accumulation of paleontological findings, along with information on extant species, made it clear that the dentitions of higher vertebrates were (1) heterodont—composed of multiple tooth types, and (2) organised into morphogenetic fields. For example, Bateson,^10^ amassed extensive data on meristic variation, including dental examples primarily of hypo- and hyperdontia. Bateson's far-ranging compilations showed that the variable dental elements occurred at the later-forming end of a field and that these terminal teeth were metrically and morphologically most variable within a field.

The Zeitgeist of developmental biology in the early 20th century was strongly influenced by Spemann's embryological organizing centers (Spemann^11^; De Robertis^12^). In 1934 Huxley and De Beer combined the study of morphology with the morphogenetic field concept in their seminal work *The Elements of Experimental Embryology*, which closely preceded Butler's^1^ description of fields and morphogenetic variations in the mammalian dentition.

Weiss^13^ was among the leaders to reaffirm the interpretative value of the field concept. Butler^1^ postulated that morphogenetic fields can account for the way in which teeth within a particular class formed meristic series, i.e., with each tooth displaying similarities to others nearby because of the influence of a common field but with graded differences because of its position. Currently these dental elements are considered to result from the repetition of ‘developmental modules’.^14,15^ Butler's phylogenetic study of the mammalian dentition was wholly observational, but he inferred several features of the changes that meshed well with the biological features of field gradients, notably (a) a size gradient within each field, (b) a gradient of morphological complexity, from the complex pole tooth to the simpler variable tooth, and (c) a gradient of variability, with less size and shape variability in the pole tooth and increasing variability away from the pole. Documenting such field gradients was important because the observations substantiated that teeth exist within morphogenetic gradients as defined by the criteria established by Huxley and De Beer^16^ and Weiss.^14^ The phylogenetic patterns do not always match the ontogenetic situations, however; while incisors have lost teeth distally-to-mesially, the number of premolars has been reduced mesially-to-distally.

Butler^17^ later suggested that teeth were evolving as a part of a system rather than as individual organs. He postulated two different effects: a ‘meristic’ effect, influencing the number and spacing of teeth, and a ‘field’ effect produced by substances or signals that controlled their differentiation and final shapes. He added that the order of development of teeth also represents a field effect.^18^

Dahlberg^2,3^ adapted Butler's^1^ concepts to the human dentition and proposed that there was a field of influence operating on each of the tooth classes, i.e., incisors, canines, premolars and molars. Each field was thought to have its strongest effect on the anterior or key tooth within a class. The more distally placed teeth, which generally develop later than those more mesially placed, were observed to show greater phenotypic variation. Interestingly, Dahlberg^2,3^ did not define fields within the primary dentition and, without any comment, he added a premolar field to Butler's^1^ three-field paradigm. In contrast, Butler^19^ later argues that premolars represent modified anterior members of a permanent molar field, with the primary second molar displaying similar morphology to the permanent first molar located directly distal to it.

Osborn^4^ proposed that a single clone of pre-programmed cells led to the development of all the teeth within a particular class. For example, a molar clone of cells was postulated to induce the dental lamina to initiate molar development. As the clone of cells grew distally, tooth buds were formed, surrounded by zones of inhibition that prevented other teeth developing until the migrating clone had moved on sufficiently. More recent work invokes a reaction-diffusion model in the region of a presumptive tooth, where activators induce placode formation while negative regulators are higher in interplacodal regions, which prevents tooth formation and, thus, accounts for the orderly spacing of teeth.^20^

The demonstration that isolated presumptive first molar tissue explants could continue to grow and form all three molar teeth in their normal sequence, provided strong experimental support for the clone model.^21^ However, the clone model does not provide an explanation for how the dentition develops as a whole, with different tooth classes displaying different shapes. Furthermore, the inclusion of the phrase “fields versus clones” in the title of Osborn's original paper^4^ has led many to suppose that the field and clone models are competing, mutually exclusive concepts. Recent findings about the roles of signalling molecules and the expression of homeobox genes during dental development indicate that such a distinction is unwarranted.^6^ Indeed, the field and clone models can be viewed as complementary to each other.

## 2 The odontogenic homeobox code

The odontogenic homeobox code model explains how dental patterns can be generated from different domains of expression of homeobox genes in neural crest derived ectomesenchyme.^5,22^ While the code was initially developed from studies of the mouse dentition in which there are only incisor and molar teeth, it has been extended to explain how canines and premolars could also be produced by overlapping domains of gene expression.^22^ Experimental studies have also shown that by modulating certain signalling molecules it is possible to alter homeobox gene expression domains in the ectomesenchymal tissue and to modify tooth number, size, shape and differentiation.^23,24^ A schematic representation of how dental patterning can be produced by an odontogenic homeobox code has been provided recently by Cobourne and Mitsiadis.^25^ They describe how an “inter-mixing” of genes expressed by ectomesenchyme of the first branchial arch can lead to the establishment of different morphogenetic fields. Patterns are established by signals from the ectoderm that induce specific domains of homeobox gene expression in the ectomesenchyme. This patterning is plastic initially but over time it becomes fixed into the ‘memory’ of the ectomesenchymal cells. It is these specific domains that are postulated to provide the molecular information needed to specify different tooth shapes.^25^

There is evidence that the nature of the molecular signalling in the upper and lower jaws may vary. The dental formula is the same in both arches in mice and in humans, but the shape and morphologies of the homologous teeth in the two jaws are each distinctive one from the other.^26^ Biochemical signalling differences have been demonstrated in the mouse for Dlx genes^27,28^ and also for activin/follistatin,^29^ although it is not known how neural crest derived cells migrating into the developing maxillary and mandibular regions develop the ability to respond differently to ectodermal signaling. Reports of apparently independent genetic determination of maxillary and mandibular dentitions, based on tooth size data derived from twins,^30^ are consistent with the molecular evidence.

## 3 Recent perspectives of the field, clone and homeobox models

Apart from the role of homeobox genes in determining the location of different fields within the developing dentition, it has been shown that expression of the signalling molecule ectodysplasin (EDA) is important in defining the size of dental fields. Studies using transgenic mice have shown that alteration in tooth number occurs when EDA, its receptor (EDAR), or the intracellular adaptor protein EDAR-binding death domain adaptor (EDARADD) are disrupted. It has been proposed that if there is over-production of the EDA ligand, or the receptors for EDA are over-expressed, then the size of the molar field in mice increases, resulting in the formation of supernumerary teeth.^31^ In transgenic mice, the supernumerary teeth develop mesial to the first molar, in the diastema region that is present normally between the incisor and molar segments. The supernumeraries usually have a reduced cusp pattern and do not resemble normal mouse molars. We would raise the question whether these ‘supernumeraries’ actually arise from tooth germs that would normally undergo apoptosis during development. In contrast to over-expression of EDAR and its association with extra teeth, loss of EDAR signalling leads to missing molars.^32^

Peterková et al.^33^ have identified five different morphotypes in the mandibular molar region of tabby/EDA mice and reported that these result from abnormal segmentation of the epithelial compartment of the dental lamina along a mesiodistal axis. Increased apoptosis has been noted in the mesial aspect of the dental epithelium of affected mice, and this seems to be a consequence of impaired tooth development associated with the defective segmentation process.^34^

In humans, apart from the X-linked form of ectodermal dysplasia (XLHED) that involves mutation of the ectodysplasin gene ED1 (locus Xq12-q13.1), autosomal forms of the condition have also been described.^35,36^ These autosomal dominant and recessive forms of HED are linked to mutations of EDAR at the 2q11-q13 locus or EDARADD at the 1q42.2-43 locus.

**Fig. 1:**
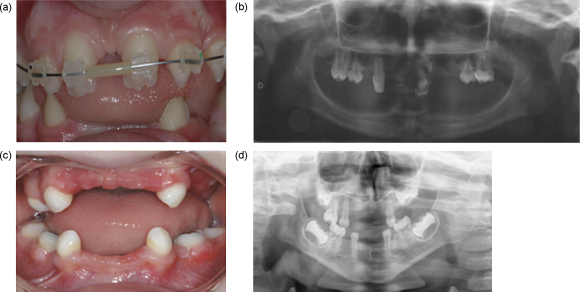
Dental phenotypes associated with an autosomal form of hypohidrotic ectodermal dysplasia (HED), linked to EDAR mutation. Clinical phenotype associated with a 10-year-old boy includes oligodontia (agenesis of mandibular permanent incisors and maxillary lateral incisors) and conical-shaped canines (a). A panoramic radiograph (b) of another patient (7 years old) with autosomal HED shows mandibular anodontia and maxillary oligodontia. There is agenesis of the primary right central incisor, lateral incisors, left canine and first primary molars with presence of only one dysmorphic permanent right central incisor germ and permanent first molars (b). Clinical (c) and radiographic (d) appearance of another patient (5 and 1/2 years old) with an autosomal dominant form of HED. Agenesis of maxillary and mandibular primary incisors, first molars and second maxillary right primary molar is evident. There is also agenesis of all permanent tooth germs, except for the permanent maxillary and mandibular first molars.

The phenotypes of autosomal dominant and recessive forms of HED, linked to EDAR mutation, are similar to the XLHED phenotype, including hypohidrosis, hypotrichosis and oligodontia, which reflects the common developmental patterns of many ectodermal derivatives.^37,20^ However, some differences are evident. The skin and hair phenotypes associated with the autosomal forms of HED seem to be milder than those in XLHED.^38^ Craniofacial dysmorphic features observed in autosomal HED also seem to be less severe than in XLHED, with mild frontal bossing and malar flattening.^35^ Moreover, dental and craniofacial phenotypic manifestations are less severe in autosomal dominant forms than in autosomal recessive HED.^36^ As for the dental phenotype of autosomal HED, hypodontia, often severe, with varying degrees of primary and permanent tooth agenesis is evident, associated with anomalous conical incisors and canines (Fig. 1).

The X-linked form of HED constitutes the majority of HED phenotypes. Nevertheless, the *ED1* mutation is not systematically identified in patients with HED: only 65.4% in a group of patients exhibiting the HED phenotype,^39^ other loci being mutated, such as *EDAR* (in 25% of non-ED1 HED) and, less often, *EDARADD*.^36,40^

Dental phenotypic variability is observed in autosomal HED (as shown in Fig. 1) and is probably due to genetic heterogeneity.^38^ Indeed, different non-sense mutations distributed along the 11 exons of the EDAR gene have been identified and are associated with varying phenotypic expression of autosomal HED.^36^ These loss-of-function mutations lead to alteration of EDAR trimerisation and partial or total loss of EDA-EDAR and EDAR-EDARADD molecular interactions.^38^ An absence or reduced activation of NF-κB during embryogenesis and abnormal cell signaling are the consequences of the EDAR mutations. The same molecular mechanisms are involved in EDARADD mutations leading to complete or partial abrogation of NF-κB activation in both autosomal dominant and recessive mutations.^40^

Line^41^ has emphasised that the concept of morphogenetic fields should not be limited to the expression of a single gene or its protein product but rather include consideration of how various genetic influences, modulated by epigenetic effects, affect development of the dentition. By studying variation in the number of missing teeth in families with mutations of the MSX1 and PAX 9 genes, Line has attempted to clarify the relative influence of these two genes within the human dentition, referring to their influences as “relative molecular morphogenetic fields”. Variations in patterns of symmetry of missing teeth within individuals were also used to gain insight into the effects of these two genes on the developmental stability of dental fields. Line found that the morphogenetic fields associated with MSX1 and PAX9 were not confined to a single tooth class, nor did there seem to be a simple gradient pattern. He interpreted these findings as reflecting an interaction between different signalling molecules. Although there was some evidence that mutations in MSX1 and PAX9 could increase the extent of asymmetry of tooth agenesis, no consistent pattern was identified. These findings are all consistent with the multifactorial aetiology as proposed by Grüneberg,^42^ Brook,^43,7^ Chosack et al.,^44^ Brook et al.^45^ and others.

**Fig. 2:**
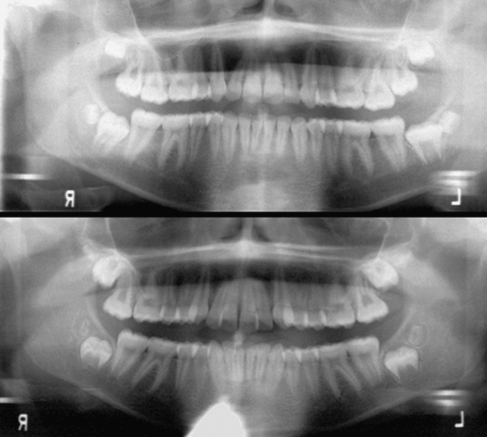
Panoramic radiographs of two American Black adolescents with a developing fourth molar in each quadrant. These supernumerary teeth are reduced in size, but exhibit differentiation into multiple occlusal cusps. These extensions of the molar fields could conceivably involve differences in these subjects’ ectodysplasin, as described by Tucker and Sharpe.^31^

Much less is known about the epidemiology or genetic factors influencing supernumerary teeth (Fig. 2). Brook^7^ proposes that the addition of “extra” teeth represents the antithesis of hypodontia. With supernumerary teeth, other teeth tend to be larger and morphologically more complex. Recent work^45,46^ also suggests that the tempo of tooth formation is depressed with hypodontia; we speculate that, conversely, supernumerary teeth may be associated with faster tempos as occurs among human ethnic groups with varying tooth sizes and morphologies.^47^ Supernumerary teeth, notably the addition of eumorphic teeth, show appreciable ethnic variation,^48^ and they tend to cluster in families. The extension of a tooth class by replicating another tooth at the variable end of tooth field gradient also corresponds to the model of reiterative signaling – repeating the engrained ‘developmental module’ used to develop the prior teeth in the same class^13,49,15^ – and may represent phenomena seen with EDA and EDAR.^31^ However, this would not immediately explain mesiodens and paramolar supernumeraries.

Mitsiadis and Smith^6^ have described a synthesis to explain dental patterning, referred to as cooperative genetic interaction (CGI), which combines the field, clone and odontogenic homeobox models. Their concept is built on evidence that neural crest derived mesenchymal cells are influenced by signals from the oral epithelium to express homeobox containing genes.^50,51^ All three elements – the “clones” of neural crest derived cells, the homeobox containing genes in the mesenchyme, and the signalling molecules released by the oral epithelium – are considered to play important roles in patterned tooth development. For example, it is proposed that mutations in genes in the mesenchymal cell clones could affect their cellular proliferation, leading either to agenesis or reduction in size of a tooth. A key feature of this model is its time-dependent nature, not only in terms of the importance of the timing of release of various signalling molecules from the oral epithelium and the subsequent diffusion gradients that are established, but also in terms of the temporal expression of different combinations of homeobox genes from the proliferating neural crest derived mesenchymal cells.

## 4 Phenotypic variation, key teeth and heritability estimates

In adapting Butler's field concept to the human permanent dentition, Dahlberg^2,3^ referred to the most stable tooth in each field as the ‘key’ or ‘pole’ tooth. These key teeth in the maxillary dentition were identified as the central incisor, the canine, the first premolar and the first molar. A similar pattern was identified in the mandibular dentition, except that the lateral incisor was identified (without any comment) as the key tooth in the incisor region. Univariate analyses of dental crown size, including variability (based on coefficients of variation) and asymmetry (based on quantitative measures of fluctuating asymmetry), have tended to conform with expected patterns.^52^ Furthermore, the observed patterns of phenotypic variation seem to be generally consistent with the relative amount of time that each developing tooth germ spends in the soft tissue phase prior to calcification. For example, the permanent maxillary incisors appear to differentiate at similar times, but initiation of mineralisation of the lateral incisor occurs later than the central whereas crown completion is slightly earlier for the lateral than the central.^53^ Therefore, the developing maxillary lateral incisor spends a relatively longer period in its soft tissue stage prior to calcification than the maxillary central incisor, during which time epigenetic and environmental factors may influence its final size and shape. Indeed, Keene^54^ has provided a useful general concept, which he referred to as the morphogenetic triangle, that describes the relationships between initiation of proliferation, initiation of differentiation and crown completion during odontogenesis. This, the dynamic development of a tooth is seen in the continued growth among formative cusps so the spatiotemporal relationships continue to change until dentinogenesis bridges the cusps and ‘petrifies’ the occlusal phenotype.

Using a multivariate statistical approach, Harris^55^ has shown that most of the variation in mesiodistal and buccolingual crown dimensions of human permanent teeth is shared, with the majority of the shared variation being associated with tooth type (i.e., whether teeth are incisors, canines, premolars and molars), and very little attributable to tooth position (i.e., ‘key’ or ‘pole’ tooth versus distal tooth within a particular class). Harris notes that his finding in relation to tooth type is intuitively appealing as it conforms with the concept of heterodonty, with four basic morphogenetic fields corresponding to incisors, canines, premolars and molars as proposed by Dahlberg. However, the data used for this analysis and for most other multivariate analyses of this type^56,57^ are traditional crown diameters, i.e., maximum mesiodistal and buccolingual diameters, and whether similar patterns would be found if other phenotypes were used, e.g., crown heights, intercuspal distances and angles, or perimeters, areas and volumes is a research question for future studies using new measurement techniques.

Similarly, studies aimed at disclosing patterns in estimates of heritabilities for dental crown size within the human dentition have also been based on mesiodistal and buccolingual crown diameters. In these studies it has generally been assumed that the key tooth within each morphogenetic dental field should display the highest heritability. In contrast, the non-key teeth, often those more distally positioned within a class, would be expected to show lower heritabilities. Some researchers have reported trends in heritability estimates that conform with this pattern^58^ but others have been unable to find any such trends.^59^

There are at least two reasons why these inconsistent findings are not unexpected. Firstly, we have seen that there is a complicated series of epigenetic and morphogenetic events involved in odontogenesis, apparently without any over-riding genetic control mechanism. Temporo-spatial variations in signalling pathways during development can lead potentially to different outcomes (or phenotypes), and it seems likely that the longer a tooth or component of a dental crown remains in its soft tissue phase, prior to crown mineralisation, the more opportunity exists for phenotypic variations to be expressed.^60,61^ In these circumstances, it is unlikely that statistically significant differences in estimates of heritability for final crown size or shape will be discerned. Secondly, most previous genetic studies of human tooth size have been based on traditional measures of overall crown size. These measures represent composites of the various components of a dental crown and are rather crude representations of complicated morphologies. Interestingly, heritability estimates for intercuspal distances of molar teeth, which may be more biologically meaningful phenotypes than overall crown measures, are only moderate in magnitude.^62^ Given that secondary enamel knots^63,64^ that form during molar odontogenesis will determine sites of future cusp tips, the moderate heritability values obtained for intercuspal distances are consistent with the important role of self-organizing, sequential epigenetic processes in enamel knot formation during odontogenesis.

## 5 Genotypes, phenotypes and developmental biology

Jernvall and Jung^49^ have highlighted the value of multiple approaches to the study of dental development, with developmental genetics, mathematical modelling, and population genetics being used to link development with both micro- and macro-evolution. They explain how the repeated activation of the same set of genes, or a so-called ‘developmental module’, accounts for the cumulative variation observed in later-developing molar cusps. Salazar-Ciudad and Jernvall^65^ have provided a mathematical model to demonstrate that the morphology of mammalian teeth can be predicted by integrating experimental data on gene interactions and growth into a “morphodynamic mechanism”, thereby linking genotype to phenotype. Jernvall et al.^66^ make use of a topographic method, Geographic Image Systems (GIS), to show that molecular pre-patterning can predict the arrangement of cusps more than a day in advance, with subtle heterotopic shifts being postulated to play an important role during evolution in producing new cuspal arrangements.

More recently, Salazar-Ciudad et al.^67^ have suggested that developmental mechanisms used for generating patterns can be grouped into three categories: cell autonomous mechanisms; inductive mechanisms; and morphogenetic mechanisms. The first category is postulated to include those mechanisms by which cells become arranged into patterns without interacting with other cells. The second category refers to mechanisms by which cells communicate with one another to produce patterning through reciprocal or hierarchical alteration of phenotypes. The third category refers to mechanisms that can lead to changes in patterns through cell interactions that do not involve phenotypic changes in cells. Of particular relevance to morphogenetic fields, inductive and morphogenetic mechanisms can be combined either morphostatically, in which the inductive mechanisms occur first, or morphodynamically, in which both types of mechanisms interact continuously. Salazar-Ciudad et al.^67^ suggest that the mammalian dentition provides a good example of a developmental system that employs morphodynamic mechanisms, with induction and morphogenesis taking place concurrently and interdependently. They also explain how morphodynamic mechanisms use spatial epigenetic information present in the emerging phenotype at each stage of the developmental process to influence later development. In this sense, morphodynamic mechanisms exhibit dependency on the so-called ‘intermediate phenotype’.

Salazar-Ciudad et al.^67^ avoid using the term ‘morphogenetic field’ because it presupposes the concept of prospective cell fate. They prefer to use the term ‘gene expression territory’. They emphasise that morphodynamic mechanisms involve interaction between cells along a developmental trajectory that is continually interacting with a changing molecular and geometric microenvironment. With this perspective of dental development, it becomes more apparent that the field and clone models are complementary interpretations of the same observations; they are mutually supportive rather than opposing concepts. This perspective is also entirely consistent with recent findings of discordant dental development in monozygotic twin pairs.^68,69^

## 6 Genetics, epigenetics and environment: the MZ co-twin model

While molecular geneticists have tended to focus on methylation and acetylation of DNA when referring to epigenetics, there is growing recognition of the need for a broader interpretation of the term.^70,71^ We use the term ‘epigenetics’ in the broad sense, as proposed originally by Waddington,^72,73^ to refer to processes by which genotype gives rise to phenotype. This broader view enables description of those interactive processes that occur between cells at the local tissue level during dental development as epigenetic events, in addition to those that may operate directly on DNA. Molenaar et al.^74^ referred to epigenetic influences as a “third source of developmental differences” that can account for phenotypic variation in development, in addition to genetic and environmental factors. This third source is comprised of non-linear epigenetic events that can cause variability at all phenotypic levels and that these epigenetic influences result from autonomous developmental processes with “emergent self-organizing properties.” The role of epigenetic factors in the broad sense during odontogenesis has been discussed by Lesot et al.^75^ while local epigenetic influences, including cell–cell and cell–matrix interactions and their effects on asymmetrical growth and differential cell proliferation during dental development have also been described.^76^

Interestingly, Eaves et al.^77^ have pointed out that Molenaar's concepts can be interpreted in terms of chaos theory. They have shown through simulations that chaotic and near-chaotic processes may reduce the strength of correlations between twin pairs. By simulating a simple non-linear model behaving chaotically, Eaves et al.^77^ found that “small variations in initial conditions (e.g., small quantitative differences between MZ twins at the molecular level such as the degree of methylation of a particular gene in a particular tissue) will have consequences at the phenotypic level which look like occasion-specific environmental effects.” The findings of Eaves and colleagues are particularly relevant when attempting to explain differences in expression of missing and extra teeth between monozygotic co-twins with the same genotypes.

Eaves et al.^77^ provide theoretical support for our view that minor variations in local epigenetic events during dental development, probably relating to the spatial arrangement of odontogenic cells and/or the timing of the signalling events between them, can produce distinct differences in dental features between monozygotic (MZ) twin pairs who have been confirmed to have the same genotypes.^67,68,78^ These differences can include discordances in the number and/or location of congenitally missing teeth or differences in the number of supernumerary teeth between MZ co-twins.

## 7 Stepping into the clinical setting

The approaches of Line^41^ and Mitsiadis and Smith^6^ provide an important step forward in explaining how the field, clone and homeobox code concepts can all be used as a foundation for understanding how phenotypic patterning is established in the human dentition. We now provide some additional perspectives based on our own research to position these new concepts within a clinical context that ranges from minor anomalies of the size and shape of teeth through to more severe anomalies, including missing and extra teeth.

The most common clinical presentation involving missing teeth is hypodontia of only one or a few teeth, commonly the third molars, second premolars or maxillary lateral incisors. On the other hand, these generalities are based on the numerous studies of peoples of European extraction,^79^ and the limited data from other ethnic groups suggest the frequencies and patterns may differ.^80–82^ When considering supernumerary teeth, the most common example is the mesiodens which occurs in the anterior midline region of the maxilla. A multifactorial model linking tooth size and number can account for the different patterns of expression of tooth size, hypodontia and supernumerary teeth observed in males and females.^7,43,45,83–85^ The relatives of individuals with missing or extra teeth have also been found to be more likely to also display missing or extra teeth, supporting the concept of an underlying genetic predisposition to hypodontia or supernumerary teeth. We believe that such a multifactorial model, with multiple genetic, epigenetic and environmental influences, provides the best explanation for our observations involving missing and extra teeth in MZ twin pairs.

Molecular studies, primarily in mice, show that congenitally missing teeth can arise from diverse developmental causes. Tooth development can be interrupted at, at least, three stages. First, the ectoderm may not have the capacity to induce bud-formation in the underlying mesenchyme.^20,86^ Second, missing or mutant signals of a mesenchymal transcriptional factor such as Msx1 or Pax9 can arrest development prior to enamel knot formation.^87^ Third, development can be arrested shortly after the initiation of dentinogenesis, with subsequent apoptosis. This latter stage corresponds to the incipient formation of lateral incisors in mice,^88,89^ where these teeth never fully develop in this species. Similarly tooth germs anterior to the first molar (i.e., premolars and canines) in mice undergo apoptosis.^34^

**Fig. 3:**
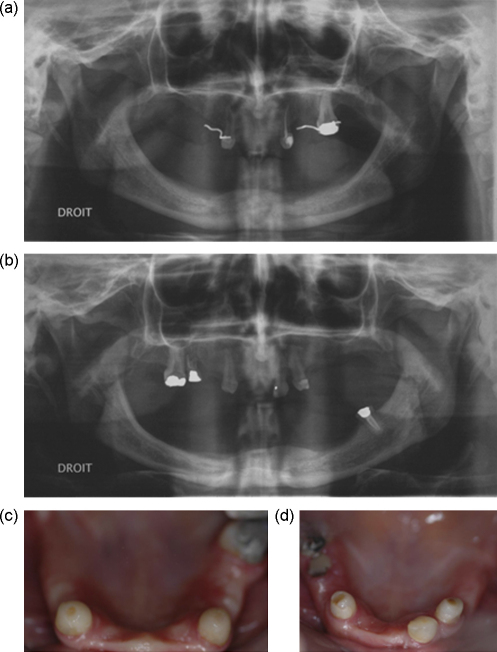
Dental panoramic radiographs (a and b) and maxillary clinical views (c and d) of a pair of monozygotic twins affected by X-linked hypohidrotic ectodermal dysplasia (XLHED) with a mutation of the ED1 gene, coding for ectodysplasin (EDA) morphogenetic factor.

To illustrate the dental variability between MZ co-twins, a pair of twins diagnosed with a mutation of the ED1 gene are described, both showing oligodontia but with different patterns of missing teeth (Fig. 3). One of the MZ twins (a) showed mandibular anodontia, whereas the other exhibited a severe mandibular hypodontia with the presence of a mandibular left permanent molar (b). The distribution of maxillary teeth again illustrates variability in the severity of the phenotype. Only permanent maxillary canines and the left first molar are evident for the first MZ twin, whereas maxillary canines and the contra-lateral right permanent first molar are present in the second MZ twin (b), together with the left maxillary central incisor and a retained maxillary second right primary molar.

The dental phenotypic variability between these XLHED co-twins could be accounted for by different local epigenetic and environmental factors interacting with the EDA mutation, as described in the multifactorial theory. The dental phenotype of the second XLHED MZ twin (b) suggests residual capacity for initiation of odontogenesis, allowing the development of a permanent maxillary central incisor and a permanent mandibular molar. However, the phenotypic variability observed shows that other epigenetic mechanisms, besides genetic factors, represented mainly by the odontogenic homeobox code and signaling molecules such as FGF, BMP, Shh, and EDA-NF-kappaB,^90,91^ are also important for initiation of odontogenesis and tooth morphogenesis.

The strong link between tooth number, size and shape is illustrated by the dental phenotype of these XLHED patients. Microdontia of canines (Fig. 3c and d), dysmorphic cone-shaped incisors (d) and canines (c and d) and molar root fusion and shape anomalies (a and b) are associated with the phenotype of oligodontia and also linked to the ED1 mutation and molecular alterations of the EDA-NF-kappaB signalling pathway.^92^

**Fig. 4:**
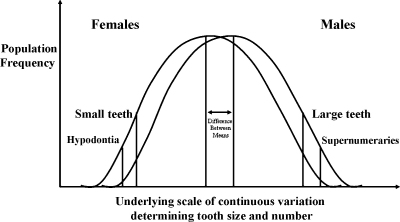
Model based on normal distributions with superimposed thresholds to explain the relationships between anomalies of tooth number and size after Brook.^7^

By superimposing thresholds on an underlying distribution of tooth size, it is possible to explain the relationship between tooth size and the presence or absence of teeth. For example, missing teeth will tend to occur below a certain threshold for tooth size. The prevalence of missing teeth will be greater in females who have smaller teeth, on average, than males. At the other extreme of the distribution, where tooth size is larger, another threshold is reached above which extra teeth will tend to occur. The prevalence of extra teeth will tend to be greater in males who have larger teeth, on average, than females. These relationships are summarised in the model of Brook (Fig. 4). It is also helpful to remember that the ‘thresholds’ actually are zones rather than well-demarcated lines because of spatiotemporal effects of the person's genetic background and dynamics of his environment.^93,94^

Although the model is a theoretical one, it has direct relevance to clinical practice. It emphases that common anomalies such as missing and extra teeth represent part of a continuous spectrum of inter-related dental phenotypes that are influenced by a combination of genetic, epigenetic and environmental factors. Furthermore, when linked to the concept of morphogenetic fields, this conceptual model enables clinicians to predict which teeth are most likely to be affected, e.g., third molars, second premolars and maxillary lateral incisors display hypodontia most commonly and are also most often affected by certain other anomalies of development. Such a framework provides a valuable basis for clinical diagnosis, treatment planning and counselling. Apart from the value to the clinician of appreciating that there are links between tooth size, shape and number within the dentition, reflecting underlying developmental processes, there are many other dental phenotypes that have been shown to be associated with congenitally missing teeth.^94^ These include delayed formation and eruption of other teeth, malposition and ectopic eruption of teeth, taurodontism, rotation of teeth, short roots and simplified morphology.^95–101^ Furthermore, more generalised anomalies in the craniofacial region have also been shown to be associated with missing teeth, including alterations in dental arch form, presence of Class II division 2 craniofacial type and sella turcica bridging.^44,102,103^ A recent report has even linked tooth agenesis to colo-rectal cancer.^104^

These several associations among size and shape of structures certainly are important clinically, but they also reflect the integrated development of skeletodental complexes, both at the molecular^41^ and phenotypic levels. Indeed, Bateson^10^ described various examples of how nearby teeth are themselves affected when there is hypo- or hyperdontia, which often parallel the situations that have occurred phylogenetically.^1,105^ Understanding co-variations can help clinically as well as in the search for common developmental etiologies.

## 8 Finding the genes for dental development: implications for future practice

To date, molecular-genetic studies in humans have concentrated mainly on locating the genes associated with missing teeth. Mutations in three genes, MSX1, PAX9 and AXIN2^104,106–109^ have been shown to be associated with familial cases of severe hypodontia (where many teeth are missing). The families selected for these studies had pedigrees consistent with an autosomal dominant mode of inheritance although variations in the number and position of teeth missing have been noted. However, there are more than 300 genes that appear to be involved in dental development,^110^ and a number of them could be candidates for missing and extra teeth. Furthermore, as we have indicated in the previous section of this review, the most common clinical presentation relating to missing teeth is simple hypodontia where only one or a few teeth are missing rather than many, so identifying the causes of common hypodontia is of clinical merit.

Given that there appears to be a link between the size and shape of teeth, and hypodontia or supernumerary teeth, we propose that there is likely to be a group of genes that exert pleiotropic effects on multiple dental phenotypes, accounting for their observed co-variation. Just how many genes are involved remains to be seen, but it is possible that it may be a relatively small number. Support for this view is provided by Kangas et al.^111^ who have shown that dental characters in mice seem to be non-independent. So too, tooth crown dimensions as well as morphological traits are positively intercorrelated within and among tooth types.^57,112^ Increasing the levels of expression of just one gene can lead to increases in cusp number, altered cusp shape and position, development of longitudinal crests on teeth, and increases in tooth number.

Rather than a simple monogenic mode of inheritance alone, we consider that a multifactorial model^7,45,83^ – with genetic, epigenetic and environmental influences – provides the best explanation for our observations involving hypodontia and supernumerary teeth in MZ twin pairs, as well as different phenotypic expressions in MZ co-twins with ectodermal dysplasia. Such a model, with superimposed thresholds linking tooth size, morphology and number, provides a plausible explanation for why MZ co-twins, who have the same genotypes, may display different dental phenotypes. The MZ twin pairs may have genotypes that place them near to a threshold for either missing or extra teeth, but variations in local epigenetic events during odontogenesis may determine on which side of the threshold they fall.

As we have mentioned previously, and as is reported in other papers in this special issue of *Archives*, measures of tooth size that have been reported most commonly in the past have been maximum mesiodistal and buccolingual crown diameters. Often these measurements have been recorded from dental casts with hand-held calipers that cannot provide accuracies better than 0.1 mm. Although there have been attempts to describe tooth size and shape using other variables, including cusp areas, perimeter and area and volumes,^60,85,113^ these types of data have rarely been used in genetic studies. With the advances in image capturing techniques, including 3D laser systems, we are now in a position to define new dental phenotypes that should give clearer insights into the nature and causes of variation in the dentition.^114^

Genome-wide association studies (GWAS) are currently being used to identify genes linked to various common diseases, including coronary heart disease, hypertension, diabetes and arthritis, and it is planned to use a similar approach to identify key genes involved in dental development using a suite of newly defined dental phenotypes in both 2D and 3D.^115^ Although very important, the genetic aspects are not sufficient to explain fully how various dental anomalies arise in individuals. This is where further exploration of epigenetic factors will be essential.

Already researchers are beginning to study epigenetic biomarkers in an attempt to explain the reasons for observed differences between MZ twin pairs.^116^ At this stage the focus is on trying to determine the extent of differences in global genomic DNA methylation levels but it is likely that more specific analyses will be developed soon. Once these approaches aimed at the level of DNA are refined further, and our understanding of the nature of the epigenetic influences at a local tissue level improves, we should be able to provide a clearer picture of how genetic, epigenetic and environmental factors influence human dental development and lead to phenotypic patterning within the dentition.

## Disclosures

*Competing interests:* None declared.

*Funding:* National Health and Medical Research Council of Australia, Australian Dental Research Foundation, National Institute of Health, USA, INSERM UMR595, Wellcome Trust, UK, National French Reference Centre for Oral Manifestations of Rare Diseases.
